# Supplementary figures and images for: Differential Metabolomics Profiles Identified by CE-TOFMS between High and Low Intramuscular Fat Amount in Fattening Pigs
Source: Metabolites. 2020 Aug 7;10(8):322. doi: 10.3390/metabo10080322 (PMC7464425; doi:10.3390/metabo10080322)

## Slide 1
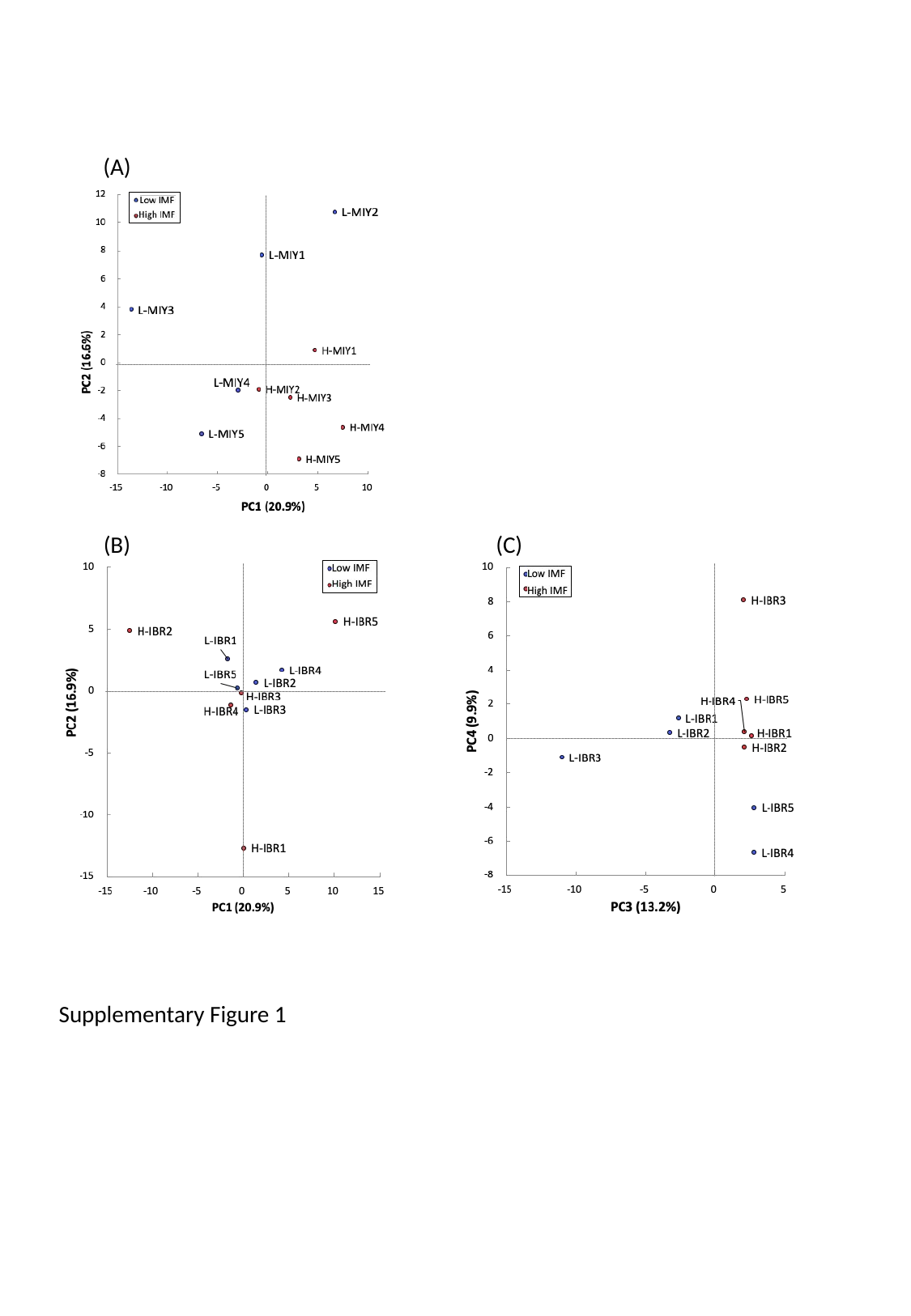

(A)
(C)
(B)
Supplementary Figure 1

Supplement: Supplementary file 1 [file metabolites-10-00322-s001.zip › FigureS1_Metabolites_MTaniguchi.pptx]
